# Different DNA methylation patterns detected by the Amplified Methylation Polymorphism Polymerase Chain Reaction (AMP PCR) technique among various cell types of bulls

**DOI:** 10.1186/1751-0147-52-18

**Published:** 2010-03-05

**Authors:** Nawapen Phutikanit, Junpen Suwimonteerabutr, Dion Harrison, Michael D'Occhio, Bernie Carroll, Mongkol Techakumphu

**Affiliations:** 1 Department of Obstetrics Gynaecology and Reproduction, Faculty of Veterinary Science, Chulalongkorn University, Henri Dunant Rd, Bangkok 10330, Thailand; 2 School of Chemistry and Molecular Bioscience, Faculty of Science, The University of Queensland, Brisbane, QLD 4072, Australia; 3 School of Animal Studies, Faculty of Natural Resources, Agriculture and Veterinary Science, The University of Queensland, Gatton, QLD 4343, Australia

## Abstract

**Background:**

The purpose of this study was to apply an arbitrarily primed methylation sensitive polymerase chain reaction (PCR) assay called Amplified Methylation Polymorphism Polymerase Chain Reaction (AMP PCR) to investigate the methylation profiles of somatic and germ cells obtained from Holstein bulls.

**Methods:**

Genomic DNA was extracted from sperm, leukocytes and fibroblasts obtained from three bulls and digested with a methylation sensitive endonuclease (*Hpa*II). The native genomic and enzyme treated DNA samples were used as templates in an arbitrarily primed-PCR assay with 30 sets of single short oligonucleotide primer. The PCR products were separated on silver stained denaturing polyacrylamide gels. Three types of PCR markers; digestion resistant-, digestion sensitive-, and digestion dependent markers, were analyzed based on the presence/absence polymorphism of the markers between the two templates.

**Results:**

Approximately 1,000 PCR markers per sample were produced from 27 sets of primer and most of them (>90%) were digestion resistant markers. The highest percentage of digestion resistant markers was found in leukocytic DNA (94.8%) and the lowest in fibroblastic DNA (92.3%, *P *≤ 0.05). Spermatozoa contained a higher number of digestion sensitive markers when compared with the others (3.6% *vs*. 2.2% and 2.6% in leukocytes and fibroblasts respectively, *P *≤ 0.05).

**Conclusions:**

The powerfulness of the AMP PCR assay was the generation of methylation-associated markers without any prior knowledge of the genomic sequence. The data obtained from different primers provided an overview of genome wide DNA methylation content in different cell types. By using this technique, we found that DNA methylation profile is tissue-specific. Male germ cells were hypomethylated at the *Hpa*II locations when compared with somatic cells, while the chromatin of the well-characterized somatic cells was heavily methylated when compared with that of the versatile somatic cells.

## Background

Methylation of genomic DNA plays an important role in genomic imprinting, X-chromosome inactivation, tissue-specific gene expression and silencing of retrotransposable elements [1]. In mammalian genome, DNA methylation occurs mainly at the cytosine residues [2] and its pattern changes according to different gene activities during cellular development [3-5]. Different genome-wide methylation content between different cell types has been investigated in murine and bovine tissues for more than 20 years [6-8]. However, the results were mostly qualitative and did not provide any possibility for further investigation of the differentially methylated locations in the samples.

Study of DNA methylation can be carried out in various ways. The digestion of genomic DNA with methylation sensitive restriction endonuclease enzymes combined with southern blot analysis is a classical method to give an overview of whole-genome DNA methylation profile, while location specific investigation can be archived by bisulfite sequencing [9]. Recently, arbitrarily-primed polymerase chain reaction (PCR), in combination with a technique called Random Amplification of Polymorphic DNA (RAPD), has been applied to study the genomic alterations in tissues, especially between cancerous and normal samples [10,11]. The powerfulness of this technique is the possibility to evaluate many genomic locations simultaneously by comparing PCR product alterations between tissue samples. Moreover, the PCR products can be retrieved for further investigation [12]. Therefore, in terms of DNA methylation investigation, comparison between genomic DNA and DNA digested with methylation sensitive enzymes could possibly provide some useful information about different methylation status at the same genomic location between two templates.

In this present study, we applied the technique called Amplified Methylation Polymorphism Polymerase Chain Reaction (AMP PCR) to compare methylation patterns between genomic- and enzyme digested DNA templates from various types of tissues. The main objective of this study was to apply AMP PCR assay to investigate the degree of difference of methylation content between somatic and germ cells by comparing the number of markers produced by the technique.

## Methods

All chemicals used in this experiment were purchased from BDH AnalaR^® ^(VWR International Ltd., Poole, England), unless stated elsewhere.

### Cell samples and DNA extraction

Samples used in this study were obtained from three Holstein bulls between 2 to 3 years old. Three types of cell samples were selected for this experiment. Sperm cells were selected as germ cell lineage, fibroblasts as versatile- and leukocytes as well-characterized somatic cell lineages. Spermatozoa were collected from fresh ejaculates and were separated from seminal plasma by centrifugation at 3000 rpm for 10 min at room temperature. Leukocytes were separated from fresh whole blood samples by centrifugation at 3000 rpm for 10 min at room temperature. Fibroblast cells were collected from monolayer cell culture originated from ear tissue explants.

Genomic DNA was extracted from leukocytes and fibroblasts using a commercial DNA extraction kit (QIAamp^® ^DNA mini kit, Qiagen, Hilden, Germany). DNA from sperm cells was extracted by treating the samples with lysis buffer containing 1% (v/v) Triton X-100 (Sigma, Steinhelm, Germany), 1 mM Deferoxamine mesylate (Sigma, Germany), 5 mM MgCl_2_, 0.32 M Sucrose and 10 mM Tris. Sperm DNA was then released from protamines by 5 M NaCl and 1 M Dithiothreitol (DTT, Roche, Mannheim, Germany) and was separated from the solution by alcohol precipitation. The genomic DNA samples were kept in TE buffer, and the concentration was adjusted to 10 to 20 ng/μl.

### Restriction enzyme digestion of the genomic DNA

The genomic DNA samples were treated with a methylation-sensitive enzyme, *Hpa*II (Invitrogen^®^, Hong Kong). The digestion solution consisted of autoclaved de-ionized water and buffer solution plus bovine serum albumin provided with the enzyme by the manufacturer. The amount of enzyme used to digest the genomic DNA, time and temperature applied to the digestion reaction were in accordance with the recommendation provided with the product. Digested DNA samples were ethanol precipitated and separated from digestion buffer by centrifugation at 13500 rpm for 30 min at 4°C. DNA pellet was re-suspended with autoclaved de-ionized water and kept at 4°C.

### Amplified Methylation Polymorphism Polymerase Chain Reaction (AMP PCR)

The PCR reaction consisted of DNA sample (genomic or digested template), *Taq *polymerase enzyme (AmpliTaq^® ^Stoffel fragment, Applied Biosystems, Branchburg, New Jersey, USA), 10 mM dNTPs mix (Invitrogen^®^), 10 μM custom-designed oligonucleotide primers (Invitrogen Custom Primers), Dimethyl sulphoxide (DMSO), PCR buffer (10 mM Tris, 10 mM KCl, 5 mM MgCl_2_) and autoclaved de-ionized water.

Thirty sets of custom-designed oligonucleotide primer were used. Each primer contained 10 base pairs: four of which were the *Hpa*II recognition site (5'-CCGG-3') and the other six bases were randomly designed (Table 1).

**Table 1:** Sequences of primers designed for AMP PCR in combination with *Hpa*II restriction enzyme treatment

Primer	Sequence (5'-3')	Primer	Sequence (5'-3')
1	TGGA**CCGG**TG	16	AAGA**CCGG**GA
2	AC**CCGG**TCAC	17	TC**CCGG**TGAG
3	AAC**CCGG**GAA	18	GAAT**CCGG**CA
4	TTC**CCGG**GTT	19	AC**CCGG**AAAC
5	TTTGC**CCGG**T	20	TG**CCGG**TTCA
6	C**CCGG**CATAA	21	AG**CCGG**GTAA
7	CAC**CCGG**ATG	22	C**CCGG**AAGAG
8	TCAGT**CCGG**G	23	CTA**CCGG**CAC
9	TG**CCGG**CTTG	24	ACCT**CCGG**TC
10	CC**CCGG**TAAC	25	CT**CCGG**ATCA
11	CAGTG**CCGG**T	26	TTT**CCGG**GAG
12	A**CCGG**CTTGT	27	AGG**CCGG**TCA
13	GT**CCGG**AGTG	28	CAA**CCGG**TCT
14	ACA**CCGG**AAC	29	CCG**CCGG**TAA
15	C**CCGG**ATGGT	30	T**CCGG**GACTC

Four bases in bold letters are the *Hpa*II enzyme recognition sequence

The PCR reaction was started at 94°C for 2 min and each cycle was as follows: 94°C 30 sec, 57°C 1 min, 56°C 1 min, 55°C 1 min, 54°C 1 min, 53°C 1 min. The cycle was repeated for 30 times plus a final extension at 72°C for 5 min. The PCR products were separated on 4% denaturing polyacrylamide gels by electrophoresis and silver stained.

### PCR marker classification and statistical analysis

**Figure 1:**
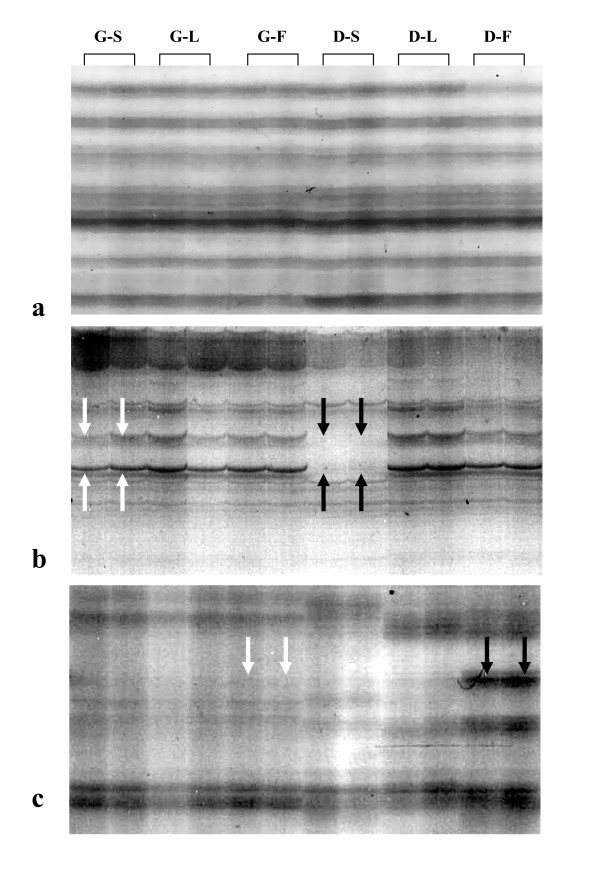
**Examples of PCR markers generated by the AMP PCR technique**. (**a**) Digestion-resistant (R) markers from primer No.21, (**b**) Digestion-sensitive (S) markers in sperm DNA produced from primer No.30 (white arrows indicate the PCR markers in the genomic samples and black arrows indicate the lost of the markers in the digested samples) and (**c**) Digestion-dependent (D) markers in fibroblastic DNA produced from primer No.1 (white arrows indicate no PCR markers in the genomic samples and black arrows indicate the PCR markers appear in the digested samples). G-S = Genomic sperm DNA, G-L = Genomic leukocytic DNA, G-F = Genomic fibroblastic DNA, D-S = Digested sperm DNA, D-L = Digested leukocytic DNA, D-F = Digested fibroblastic DNA

The comparison of markers was made based on the presence-absence manner of the PCR products between genomic and *Hpa*II digested templates. There were 3 types of markers:

(1) *Digestion-resistant (R) marker *appears both in genomic and digested DNA template (Fig. 1a), indicating that the location is resistant to the enzymatic digestion by the protection of the methylation.

(2) *Digestion-sensitive (S) marker *appears only in the genomic DNA template but not in the digested one (Fig. 1b), indicating that the enzyme can break the DNA at this location. Therefore, this location is non-methylated, and

(3) *Digestion-dependent (D) marker *appears only in the digested DNA template (Fig. 1c). The formation of this marker is still under investigation.

The observation of marker pattern was done by placing the dried silver-stained gel attached on the glass plate on a light box designed for X-ray film examination. Only clear and reproducible marker bands were counted, and the comparison of bands between genomic and digested templates was done according to the appearance of the marker mentioned above.

Number and percentage of each marker were reported in the individual bull and the average and standard deviation were calculated from the pooled data. The difference between each marker type in somatic and germ cells was evaluated by Chi-square test using a SAS statistical program (SAS 2002, SAS/Stat^®^, Cary, NC, USA).

## Results

**Figure 2:**
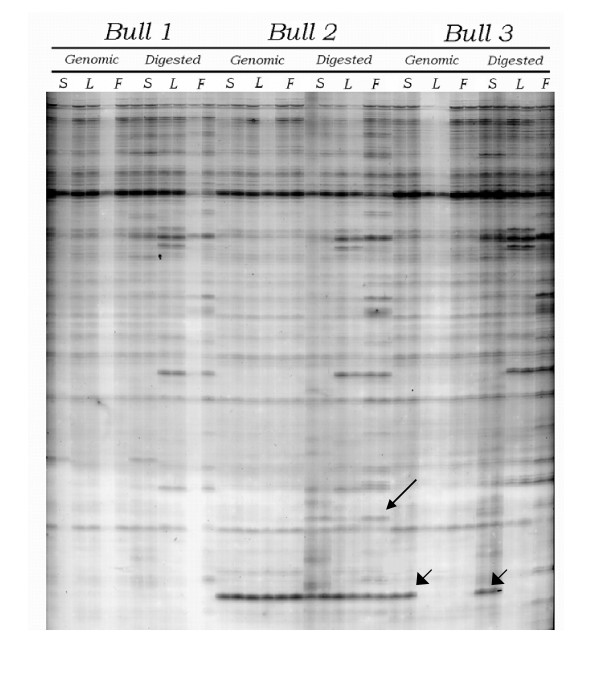
**Example of the AMP PCR profile generated by the AMP PCR technique**. This profile belonged to primer No.15. S = Sperm, L = Leukocyte, F = Fibroblast. Long arrow indicates the digestion dependent marker appeared only in bull number 2. Short arrows indicate the digestion resistant marker found in every cell sample from bull number 2 and in sperm DNA sample from bull number 3.

Cell samples from three bulls showed similar methylation profiles (Fig. 2). Approximately 1,000 PCR markers per sample per animal were produced from 27 sets of primer (Tables 2, 3 and 4) or, in average, 30-40 markers per primer. The other 3 primers gave poor marker patterns (smear or faint bands) and were excluded from the study.

**Table 2:** Summary of the AMP PCR markers found in sperm DNA

			Bull 1	Bull 2	Bull 3	Ave ± SD
Sperm DNA	R marker	n	990	997	994	993.7 ± 3.5
		%	92.8	92.9	94.4	93.4 ± 0.9
	S marker	n	37	44	34	38.3 ± 5.1
		%	3.5	4.1	3.2	3.6 ± 0.5
	D marker	n	40	32	25	32.3 ± 7.5
		%	3.7	3.0	2.4	3.0 ± 0.7

Total marker			1067	1073	1053	1064.3 ± 10.3

**Table 3:** Summary of the AMP PCR markers found in fibroblastic DNA

			Bull 1	Bull 2	Bull 3	Ave ± SD
Fibroblastic DNA	R marker	n	994	1006	1000	1000.0 ± 0.6
		%	92.3	91.3	93.3	92.3 ± 1.0
	S marker	n	27	35	24	28.7 ± 5.7
		%	2.5	3.2	2.2	2.6 ± 0.5
	D marker	n	56	61	48	55.0 ± 5.6
		%	5.2	5.5	4.5	5.1 ± 0.5

Total marker			1077	1102	1072	1083.7 ± 16.1

**Table 4:** Summary of the AMP PCR markers found in leukocytic DNA

			Bull 1	Bull 2	Bull 3	Ave ± SD
Leukocytic DNA	R marker	n	1000	1012	1004	1005.3 ± 6.1
		%	94.9	94.2	95.2	94.8 ± 0.5
	S marker	n	21	29	20	23.3 ± 4.9
		%	2.0	2.7	1.9	2.2 ± 0.4
	D marker	n	33	33	30	32.0 ± 1.7
		%	3.1	3.1	2.8	3.0 ± 0.2

Total marker			1054	1074	1054	1060.7 ± 11.5

**Figure 3:**
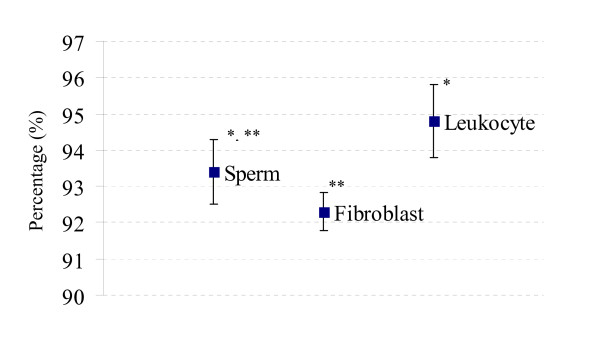
**Percentage of the digestion resistant (R) markers calculated from the pooled data**. The box represents the average percentage and the error bars standard deviations. Samples with different number of asterisk (*) are statistically different.

When the data from three bulls was pooled together and comparison was made between each cell lineage. More than 90% of markers were digestion-resistant (R) markers and the average percentage of this marker found in each sample is reported, with the error bars, in Fig. 3. Within the somatic cell lineage, a higher number of R markers were found in leukocytic DNA when compared with fibroblastic DNA (94.8% vs 92.3%, *P *< 0.05), while the amount of this marker in germ cells was in between (93.4%).

**Figure 4:**
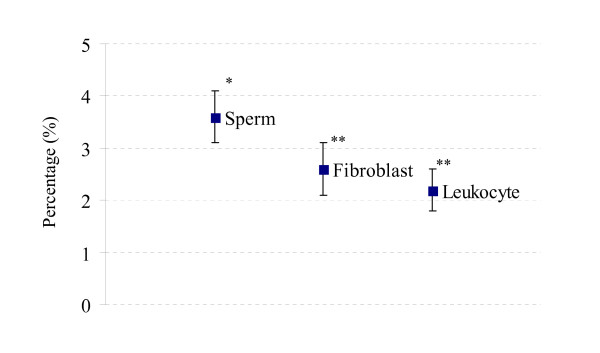
**Percentage of the digestion sensitive (S) markers calculated from the pooled data**. The box represents the average percentage and the error bars standard deviations. Samples with different number of asterisk (*) are statistically different.

The average percentage of S marker found in each sample is shown in Fig. 4. Sperm DNA significantly contained more S marker (3.6%, *P *< 0.05) than fibroblastic DNA (2.6%), and leukocytic DNA showed the lowest percentage of this marker (2.2%).

**Figure 5:**
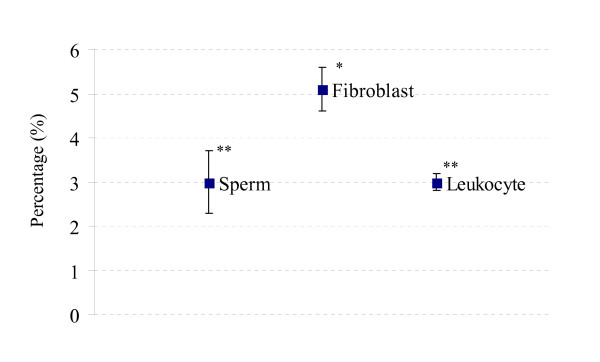
**Percentage of the digestion dependent (D) markers calculated from the pooled data**. The box represents the average percentage and the error bars standard deviations. Samples with different number of asterisk (*) are statistically different.

The highest number of the D marker was found in fibroblastic DNA (5.1%, *P *< 0.05) when compared with that in leukocytic (3.0%) and sperm DNA (3.0%) (Fig. 5).

**Figure 6:**
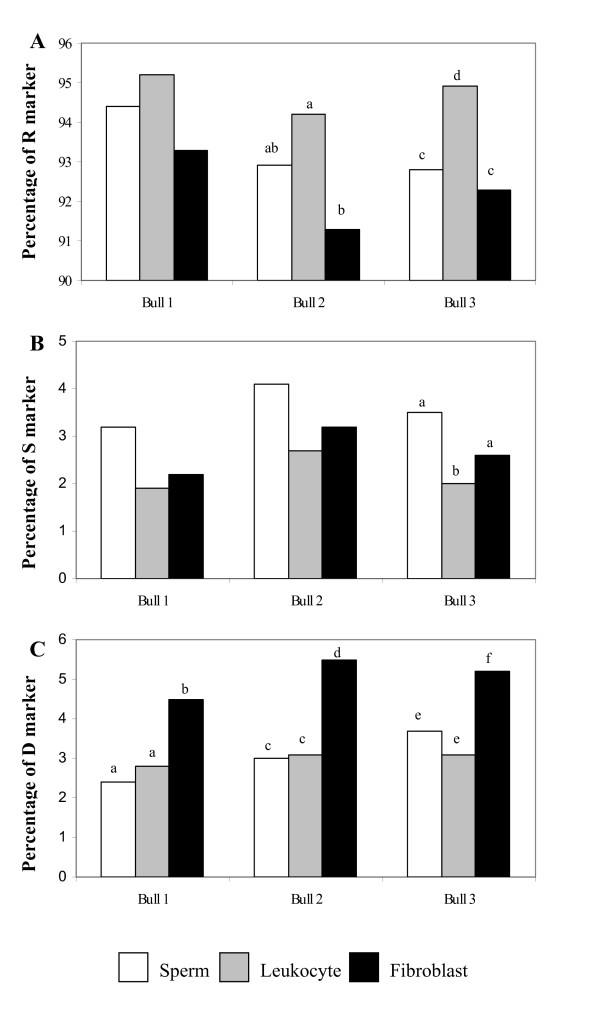
**Individual variations of markers among bulls**. Percentages of the R markers (Fig. 6-A), S markers (Fig. 6-B) and D markers (Fig. 6-C) in sperm, leukocytic and fibroblastic DNA found in each bull. Different letters between cell samples within the same bull indicate that the difference is statistic significance (*P *< 0.05).

When considered the data obtained from each bull, variations among individual were apparent. Bull No.1 had only one significant difference in the percentage of D marker between fibroblasts and the others. Bull No. 2 showed significant differences in the percentage of R marker between leukocytes and fibroblasts and in the percentage of D marker between fibroblasts and the others. While Bull No. 3 exhibited significant differences in all markers: R marker between leukocytes and fibroblasts, S marker between leukocytes and the others and D marker between fibroblasts and the others. The summary of the individual variations mentioned above is shown in Fig. 6.

## Discussion

The AMP PCR is a PCR based technique that we applied to study DNA methylation profiles in different cell types. Like other DNA fingerprinting techniques, such as RAPD or DNA Amplification Fingerprinting (DAF), AMP PCR can generate DNA markers by mean of arbitrary amplification with single short oligonucleotide primers and the resulting markers can be evaluated by electrophoresis separation on polyacrylamide sequencing gels. The genomic DNA digestion with methylation sensitive endonuclease and the use of primers containing recognition sequence of the applied enzyme that allows assessing of DNA methylation status of the particular locations throughout the genome. The alterations of PCR products in the digested DNA template provide the impression that the locations are intact or destroyed after the enzymatic digestion. The absence of PCR marker in the digested template referred to the loss of the particular genomic location due to enzymatic treatment. Therefore, this particular location is unmethylated. On the other hand, no change in PCR marker between the two templates indicates that the amplified locations are protected from the digestion by DNA methylation.

The results showed that AMP PCR assay could produce DNA marker patterns from genomic and digested DNA templates. The number of markers gained by this technique was, in average, 30-40 markers per primer, which was comparable with other studies [13,14]. In this study, we applied a high concentration of oligonucleotide primers (10 μmol) and used DNA polymerase Stoffel fragment as some reports suggested that more PCR markers could be obtained via this condition [13,15]. However, there are other factors affecting the marker production. The sequence of primer, for instance, might play an important role in this assay. From 30 sets of primer, we could summarize the results from only 27 sets, while the other three primers gave poor patterns that could not be scored. The annealing temperature in the PCR step is also crucial [16]. In the present study, we employed a high annealing temperature (53-57°C) to prevent spurious amplification. The same condition has been used in arbitrarily primed PCR technique with good marker patterns [13,17,18]. However, the amount of DNA markers gained per primer in this study was slightly low when compared with other reports. This might be due to different marker detection methods. We used acidic silver staining which has less sensitivity than radioactive or fluorescent detection.

The similar AMP PCR profiles generated from three bulls indicated that bull genome is highly conserved with approximately 1.6% variations among individuals. When the comparison of DNA methylation profiles was made between germ- and somatic cells, we found that germ cells contained less methylated *Hpa*II locations in their genome. This finding was in accordance with other reports [7,19]. The hypomethylation status of spermatozoa might be associated with a special genome structure designed for meiosis division, and possibly be involved in specific gene expression at early stage of embryo development [20].

Furthermore, when we compared the methylation patterns obtained from leukocytic and fibroblastic DNA, the results showed that leukocytes had the highest amount of DNA methylation in their genome. This result was in agreement with the knowledge that well-characterized differentiated cells need only a small number of genes to be actively expressed to maintain their functions, and the rest are suppressed by DNA methylation or other gene regulation processes [21]. On the other hand, somatic cells possessing the ability to change their morphology and cell functions like fibroblasts exhibited differently. Our results showed that fibroblast DNA was somehow hypomethylated when compared with leukocyte and sperm DNA. Moreover, we found a high percentage of digestion dependent markers in this cell type. The formation of this marker by AMP PCR technique is not clearly understood, but we hypothesized that the enzyme digestion might remove some secondary structures of the genome, and this allowed the binding of primers to their intact recognition locations hidden inside those complex structures. In this case we surmised that fibroblast cells possibly had special genomic architectures owing to their versatility. It is challenging to figure out the origin of the digestion dependent marker and the hypothesis of the complex structures could be elucidated.

From this work, we proved that the AMP PCR technique could generate methylation-associated fingerprints from different cells and tissues obtained from Holstein bulls. The technique could be used as a screening test for the DNA methylation pattern of the animal. The difference of the AMP PCR patterns between each cell type, though at a very low degree and could not be used as an individual identification tool, could possibly facilitate the discovery of some differentially methylated locations in the genome. However, the results of this present study were from the *Hpa*II enzyme recognition locations only. These particular locations are abundant in the mammalian genome and many may not closely associate with gene regulatory domains. To enhance the ability of the AMP PCR in the study of the gene-specific methylation profile in different tissues, other methylation sensitive restriction endonuclease enzymes recognizing the methylation locations within genes or gene promoter regions could provide valuable information in terms of methylation-associate gene expression. Radio-labeling or fluorescent deoxynucleoside triphospate could also be used in the PCR to increase the sensitivity of marker detection.

## Conclusions

We applied an arbitrarily primed PCR-based technique, Amplified Methylation Polymorphism Polymerase Chain Reaction (AMP PCR), to investigate DNA methylation profiles in three different cell types obtained from Holstein bulls. The methylation status of approximately 1,000 *Hpa*II locations throughout the genome could be identified by this present technique. We found that the *Hpa*II DNA methylation profile is tissue-specific. Male germ cells were hypomethylated at the *Hpa*II locations when compared with somatic cells, while the chromatin of the well-characterized somatic cells was heavily methylated when compared with that of the versatile somatic cells.

## Competing interests

The authors declare that they have no competing interests.

## Authors' contributions

NP carried out the AMP PCR assays and marker analysis. JS contributed in preparing the chemicals used in the experiment. DH and BC contributed in the experimental designs and techniques. MO and MT provided the concept of the experiment and helped to draft the manuscript. All authors read and approved the final manuscript.
